# Intercultural Competency in Public Health: A Call for Action to Incorporate Training into Public Health Education

**DOI:** 10.3389/fpubh.2015.00210

**Published:** 2015-09-02

**Authors:** Julia M. Fleckman, Mark Dal Corso, Shokufeh Ramirez, Maya Begalieva, Carolyn C. Johnson

**Affiliations:** ^1^Maternal Child Health Leadership Training Program, Department of Global Community Health and Behavioral Sciences, Tulane University School of Public Health and Tropical Medicine, New Orleans, LA, USA

**Keywords:** cultural competency, cultural diversity, public health training and education, intercultural studies, schools of public health

## Abstract

Due to increasing national diversity, programs addressing cultural competence have multiplied in U.S. medical training institutions. Although these programs share common goals for improving clinical care for patients and reducing health disparities, there is little standardization across programs. Furthermore, little progress has been made to translate cultural competency training from the clinical setting into the public health setting where the focus is on population-based health, preventative programming, and epidemiological and behavioral research. The need for culturally relevant public health programming and culturally sensitive public health research is more critical than ever. Awareness of differing cultures needs to be included in all processes of planning, implementation and evaluation. By focusing on community-based health program planning and research, cultural competence implies that it is possible for public health professionals to completely know another culture, whereas intercultural competence implies it is a dual-sided process. Public health professionals need a commitment toward intercultural competence and skills that demonstrate flexibility, openness, and self-reflection so that cultural learning is possible. In this article, the authors recommend a number of elements to develop, adapt, and strengthen intercultural competence education in public health educational institutions.

## Introduction

Cultural diversity training, most commonly labeled “cultural competency” training, has proliferated in United States health professional training institutions throughout the past decade. Cultural competence has gained attention from healthcare policymakers, professionals, and educators as a strategy to improve quality and outcomes in health care (1). This, in large part, is due to an increase in race, ethnic, and cultural diversity within the U.S. as well as standards and concomitant mandates from accrediting bodies (1–3). Several associations, including the Association of American Medical Colleges (4, 5), the Association of School of Public Health (5), the American Association of Colleges of Nursing (6), and the National Association of Social Workers (7), have all published reports with recommendations for implementing cultural competency training in educational institutions. Additionally, standards have been developed, such as the National Standards on Culturally and Linguistically Appropriate Services developed by the Office of Minority Health (8). Despite agreement to the importance of cultural competency training, a lack of conceptual clarity exists particularly in the context of educating health professionals (9, 10). Accreditation requirements across specialties remain general and highly variable (11). Further, in spite of many calls to action, a lack of consensus remains for implementation and evaluation of training curricula.

In order to be effective in a multifaceted, ever-changing public health environment, students training to be public health professionals need to develop leadership skills like the ability to engage in a process of self-reflection and awareness (12). In order to accomplish this, public health training institutions must take a diversity-based approach to organizational structure and learning (12). One way to accomplish this is by incorporating cultural competency training into a training institution’s curriculum. Public health training institutions have been more limited in their incorporation of cultural competency training, and to date there is no literature published in this area. Cultural competency also implies a one-sided approach in which it is possible to substantially know another culture, and is based on self-perceived measures, which can be unreliable. An alternate competency-based education approach, known as the intercultural competence (ICC) framework (13, 14), is more appropriate in the context of public health training institutions for fostering the development of more process-oriented learning and ultimately the growth of public health students in a leadership capacity. This model focuses more on dual cultural understanding and interaction between professionals and patients and their communities, rather than on a one-sided competence. This model will allow emerging public health professionals to gain the leadership skills to address challenges in the field, especially the increasingly complex solutions and decreased funding for population health issues.

An urgent need exists for intercultural competency public health programming and research that are conducted with an awareness of cultural differences (15). This type of training is particularly important for developing professionals who will work in health research and will develop, implement, and evaluate preventative programming. Incorporating cultural diversity training into public health curricula presents opportunities to improve programming, research, and policy around health disparities from a preventative approach. The ability to engage with diverse communities in the practice of intercultural competency enhances the likelihood that programs, services, and policies will be relevant and can prevent further health disparities.

## History of Human Subjects Research in Public Health

Barriers to cultural understanding include stereotyping, prejudice and racism, ethnocentrism, cultural imposition, cultural conflict, and cultural shock. People are frequently unaware of their own stereotyping and prejudices. An absence of cultural understanding occurs when research and programs are imposed on communities without collaborative input. In human subjects research, there is a history of exploitation of research subjects and a lack of community input both in the U.S. and internationally (16–20). However, the movement toward community-based participatory research (CBPR) has been viewed as a positive step (21, 22). CBPR is a collaborative approach that enables community members to participate in an active manner in the research process from conception to design, implementation, analysis, interpretation, conclusions, and dissemination. The goal of CBPR is to influence change in community health, systems, programs, or policies (23). Although CPBR is believed to be taking us in the appropriate direction, the need still exists for additional cultural diversity training in order to implement CPBR in the most effective way.

## Intercultural Competency vs. Cultural Competency as a Conceptual Framework

A variety of fields have conducted studies related to cultural understanding and competencies. These studies include different definitions according to interpretations of the researchers. No agreement has been reached on how concepts associated with cultural understanding and competencies should be defined (13, 24, 25). Deardorff argued that the lack of specificity in defining cultural understanding and competence is due to the difficulty of identifying the specific mechanisms of the concepts (13) and could account for the lack of consensus and/or standardization for intercultural competency training in public health, as well as in other professional areas.

The Cultural Competence Model has emerged as the primary conceptual framework for teaching cultural awareness to medical trainees. This model focuses on knowledge- and attitudes-based programming around health disparities, and on improving provider awareness of the impact of sociocultural factors on patients’ values and behaviors. A skills-building component is also built into this model to provide trainees with the opportunity to learn communication techniques to improve provider-patient communication and, ultimately, to improve patient care (26–29). Several educational frameworks and strategies have been proposed to more fully integrate cultural competency into medical, physician’s assistant, nursing, mental health provider, health education, and social work curriculums (27, 30–40). However, no standard cultural competency curriculum for health professionals exists, and a wide variety of strategies have been implemented from informal curricula that includes one short educational session to a formal full-scale integrated curriculum that is implemented over several years of study (41). Evaluation of cultural competency education shows promise in improving the intermediate outcomes of knowledge, attitudes, and skills of health professionals in dealing with patients and their communities (42–46).

Many have argued that the term “competence” is problematic because the knowledge, attitudes, and skills for both personal and professional performance that are considered have been increasingly linked with something that can be substantially learned, rather than fostering the development of process-based meaningful and transferable learning (15, 47). Cultural competency infers a one-sided approach in which it is possible to substantially know another culture. We can never become fully competent in another’s culture and heritage. Additionally, while evaluation of cultural competence education has tended to rely on participant self-assessments, research has shown that these may not be reliably associated with objective outcome measures specific to knowledge and culturally competent behavior (48–50). Cultural competence as a model demonstrates a lack of knowledge of the context and processes that influence health and healthcare of diverse patients and communities, suggesting gaps in the curricula in delivering contextual knowledge. Cultural humility, which encourages a lifelong process of self-reflection and self-critique, is now seen as a construct of a more process-oriented cultural competence approach (51, 52). This construct has been encouraged and used in training for healthcare professional students (52–55), but still focuses on provider learning and lacks an emphasis on intercultural learning and interaction. A more appropriate approach may be to use a more continuous process of self-evaluation, self-critique, and most importantly, intercultural interaction.

The ICC Model is a more appropriate educational framework in this context as it is more focused on process-oriented learning. The model allows for greater levels of shared understanding for all participants, incorporating a more systematic approach to performance progress for students in cultural understanding, and integrates an explicit teaching and learning process within a training institution. The ICC model is iterative and illustrates the movement from the personal level to the interpersonal level of interactions. This model is based on a working definition of intercultural competence: “the ability to communicate effectively and appropriately in intercultural situations based on one’s intercultural knowledge, skills and attitudes” and the elements of intercultural competence agreed on by intercultural scholars (13). As shown in Table 1, Deardorff (13) used a modified Delphi technique to develop a consensus on the definition and key components of intercultural by 13 leading national and international intercultural scholars (13).

**Table 1:** **Intercultural competence elements with 80–100% agreement among top intercultural scholars^a^**.

**Intercultural competence**
Ability to communicate effectively and appropriately in intercultural situations based on one’s intercultural knowledge, skills, and attitudes
Ability to shift frame of reference appropriately and adapt behavior to cultural context: adaptability, expandability, and flexibility of one’s frame of reference/filter
Ability to identify behaviors guided by culture and engage in new behaviors in other cultures, even when behaviors are unfamiliar give a person’s own socialization
Behaving appropriately and effectively in intercultural situations based on one’s knowledge, skills, and motivation
Ability to achieve one’s goals to some degree through constructive interaction in an intercultural context
Good interpersonal skills exercised intercultural: the sending and receiving of messages that are accurate and appropriate
Transformational process toward enlightened global citizenship that involves intercultural adroitness (behavioral aspect focusing on communication skills), intercultural awareness (cognitive aspect of understanding cultural differences), and intercultural sensitivity (focus on positive emotion toward cultural difference)
**Specific components of intercultural competence**
Understand other’s worldview
Culture self-awareness and capacity for self-assessment
Adaptability and adjustment to new cultural environment
Skills to listen and observe
General openness toward intercultural learning and the people from other cultures
Ability to adapt to varying intercultural communication and learning styles
Flexibility
Skills to analyze, interpret, and relate
Tolerating and engaging ambiguity
Deep knowledge and understanding of culture (one’s own and others’)
Respect for other cultures
Cross-cultural empathy
Understanding the value of culture diversity
Understanding of the role and impact of culture and the impact of situational, social, and historical contexts involved
Cognitive flexibility
Sociolinguistic competence (awareness of relation between language and meaning in societal context)
Mindfulness
Withholding judgment
Curiosity and discovery
Learning through interaction
Ethnorelative view
Culture-specific knowledge and understanding host culture’s tradition

*^a^Deardorff (13). Copyright 2006 by Copyright Holder*.

The ICC model illustrates movement from internal outcomes, characterized by the individual’s attitudes and attributes, to the interactive external outcomes, characterized by appropriate and effective communication and behavior in intercultural situations (13, 14). The model exhibits the continual process of development of intercultural competences. Additionally, the model emphasizes the importance of attitudes and understanding, and knowledge and comprehension. Attitude is the most vital component because it is the starting point in the process demonstrated in the model. Specifically, “attitudes of openness, respect (valuing all cultures), curiosity, and discovery (tolerating ambiguity) are viewed as fundamental to intercultural competence” (13, 14). Beyond knowledge and comprehension, skills for attaining and processing knowledge about one’s own culture as well as others’ cultures are also considered critical in developing the internal outcomes specified in the model. Such skills include listening, observing, evaluating, analyzing, interpreting, and relating. By focusing on the components of intercultural competence and how to develop them, the model provides a foundation for the general assessment of intercultural competence and also allows for the conceptualization of specific assessment indicators within a context or situation.

## A Framework for Developing Cultural Diversity Training Based on the ICC Model

**Figure 1:**
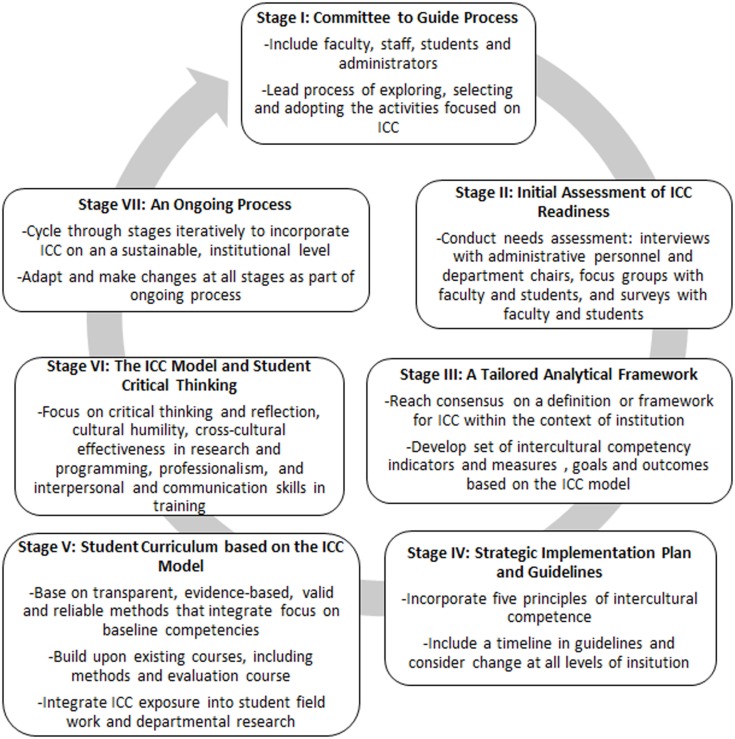
**Overview of intercultural competence (ICC) framework for Public Health Institutions**.

Based on the ICC model, we developed a framework, as shown in Figure 1, to incorporate intercultural competency training into public health institutions.

### Stage I: A committee to guide the process

The creation of a committee of selected faculty, staff and administrators is necessary to guide the process of intercultural competency integration. This committee will lead the process of exploring, selecting, and adopting the activities focused on intercultural competency. The committee will spearhead the effort to assess institutional readiness by interviewing colleagues and administering surveys. Further, they can pursue faculty and administrative buy-in by training a group faculty and staff on intercultural competence to build support. This committee should also create a strategic plan for staff and student intercultural competency training and delegate the development of an appropriate student curriculum tailored to each department. Without a carefully selected body of individuals to champion the change, the process could very well be overlooked.

### Stage II: Initial assessment of intercultural competence readiness

Faculty and students must be canvassed relative to their readiness to adopt intercultural competency training. Understanding the positive and negative influences currently in place at an institution that will hinder or support efforts to incorporate intercultural competence into the curriculum may inform the process for incorporating intercultural competency training into the institution. The institutional committee may then create a strategic plan that will most effectively drive efforts. This step should include a needs assessment of the institution, with interviews with administrative personnel and department chairs, as well as focus groups with faculty and students. The goal is to gain insight on current institutional practices, possible school wide diversity efforts, barriers to change, and assets to use in incorporating change. Surveys are also an important tool in measuring knowledge and attitudes regarding intercultural competence among faculty and students.

### Stage III: A tailored analytical framework

An institutional curriculum must be developed, adapted, and implemented in a way that is individualized to that institution. To accomplish creation and adaptation of such a curriculum, the institutional committee should develop an analytical framework, as well as reach consensus on a definition or framework for intercultural competence within the context of the institution. The framework must include a developed set of intercultural competency indicators and measures as well as goals and outcomes based on the ICC model. The analytical framework ought to address the relationship between the resources, activities, and outcomes of adaptation of an intercultural competency curriculum.

### Stage IV: Strategic implementation plan and guidelines

A strategic implementation plan to incorporate intercultural competency into the institutional training curriculum is necessary to ensure adaptation and continued improvement. The plan should incorporate five principles of intercultural understanding that include: valuing diversity, conducting self-assessment, managing the dynamics of difference, acquiring and institutionalizing cultural knowledge, and adapting to diversity and the cultural contexts of the communities served. Specific guidelines based on this plan may contain a timeline and should consider (1) the organizational structure and how to incorporate further staff and student diversity, (2) the development of an educational curriculum and materials for students and faculty, (3) incorporating methods of ICC into the institution’s research and program practices, (4) local community engagement, and (5) the evaluation of efforts.

### Stage V: Student curriculum based on the ICC model

Student curriculum must be developed based on the ICC model and tailored to institutional and community needs. The ICC conceptual model provides general guidance while the institution and community needs can be integrated to produce an effective plan (56). The plan should be based on transparent, evidence-based, valid, and reliable methods that integrate a focus on baseline competencies (57). CBPR can provide a focus for curriculum development because it includes the importance of context – for example, socio-economic, political, environmental, and cultural histories of a population. Faculty, students, and the local community play an integral role in the process of developing a strong intercultural competency training curriculum. One recommendation is that the development of a comprehensive and inclusive definition of cultural diversity – with consideration of race, ethnicity, class, age, gender, sexual orientation, disability, language, religion, and other constructs of diversity – not only be the initial step in the process of curriculum development but also be the initial and defining concept for the curriculum itself. The curriculum needs to build upon existing courses, such as methods and evaluation courses. Additionally, the curriculum should incorporate intercultural competency concepts into student field training and departmental research. The institutional committee of faculty and administrators that are both informed in intercultural competency training and responsible for school curriculum must drive the development of this curriculum.

### Stage VI: The ICC model and student critical thinking

Cultural beliefs and values vary across groups and are constantly in flux. Therefore, intercultural competence training should focus on critical thinking and reflection, cultural humility, cross-cultural effectiveness in research and programming, professionalism, and interpersonal and communication skills (58, 59). Critical thinking and reflection refer to one’s ability to identify central issues and assumptions related to intercultural competency, to remain open-minded, to analyze and synthesize information, and to evaluate and reflect on one’s own actions and the actions of those to which one is influenced. This kind of critical thinking and reflection, as related to intercultural competency training, will help build stronger public health professionals who have the potential to impact population health, providing a much-needed expansion from patient care into the community at large.

### Stage VII: An ongoing process

Training efforts are evolutionary. Institutions may begin simply by adding intercultural competency training as a specific area of study or as a component of foundational coursework, with the expectation of incorporating a more intricate, cohesive, and in-depth approach to cultural diversity in later stages. Students are expected to progress in their understanding the complexities of cultural diversity as they relate to public health and specifically populations of interest. Development of further strategic plans and a committee to guide the process are fundamental. Changes can be made in the logic model and curriculum throughout stages of development.

### Stage VIII: Evaluation and modification

Just as instructional programs and student learning are continually assessed in order to further the ongoing development of educational programs, the incorporation of cultural effectiveness into the curriculum should be evaluated as a component of the ongoing process. This constant assessment and re-assessment process, integral to public health, will provide public health training institutions with an opportunity to be consistently improving their efforts to incorporate effective community-engaged research and practice. Evaluation would include both process and outcome evaluation assessment tools, including surveys with faculty and students to assess fidelity and reach of curricula incorporation as well as changes in knowledge, attitudes, and skills in intercultural competence; and focus groups and interviews with faculty and students to gain further insight into changes in outcomes and feedback for continued improvement in training efforts. Surveys and qualitative evaluation should measure change in the intercultural competency indicators and outcomes defined in Stage 3. Results from evaluation may be used to update the strategic implementation plan and guidelines, as well as the student curriculum.

## Conclusion

Methods to incorporate intercultural competency training into public health training institutions call for a more community-engaged active approach that is integrated at multiple institutional levels. The above recommendations are founded in cultural diversity educational theory, and the ICC Model allows for adaptation to an individual institution’s own specific needs. Although it may be challenging to achieve support for incorporation of intercultural competence training, building a multilevel curriculum, and creating more cultural diversity within a public health training institution, this approach is more likely to produce positive long-term outcomes for faculty, students, and communities engaged in health research and programming. As efforts to incorporate intercultural competency training are pursued, outcomes-based evaluation and research must take place to determine the value and effectiveness of these approaches in reducing health disparities.

## Author Contributions

JF conceptualized and designed the manuscript, created the framework, drafted the initial manuscript, and approved the final manuscript as submitted. SR and MD contributed to the initial manuscript, reviewed and revised the manuscript, and approved the final manuscript as submitted. MB and CJ contributed to the framework for the manuscript, reviewed and revised the manuscript, and approved the final manuscript as submitted. All authors approved the final manuscript as submitted and agree to be accountable for all aspects of the work.

## Conflict of Interest Statement

The authors declare that the research was conducted in the absence of any commercial or financial relationships that could be construed as a potential conflict of interest.
